# Sequencing of the complete mitochondrial genome of the common raven *Corvus corax* (Aves: Corvidae) confirms mitogenome-wide deep lineages and a paraphyletic relationship with the Chihuahuan raven *C*. *cryptoleucus*

**DOI:** 10.1371/journal.pone.0187316

**Published:** 2017-10-30

**Authors:** Arild Johnsen, Anna M. Kearns, Kevin E. Omland, Jarl Andreas Anmarkrud

**Affiliations:** 1 Natural History Museum, University of Oslo, Oslo, Norway; 2 Department of Biological Sciences, University of Maryland, Baltimore County, Baltimore, Maryland, United States of America; National Cheng Kung University, TAIWAN

## Abstract

Previous studies based on single mitochondrial markers have shown that the common raven (*Corvus corax*) consists of two highly diverged lineages that are hypothesised to have undergone speciation reversal upon secondary contact. Furthermore, common ravens are paraphyletic with respect to the Chihuahuan raven (*C*. *cryptoleucus*) based on mitochondrial DNA (mtDNA). Here we explore the causes of mtDNA paraphyly by sequencing whole mitochondrial genomes of 12 common ravens from across the Northern Hemisphere, in addition to three Chihuahuan ravens and one closely related brown-necked raven (*C*. *ruficollis*) using a long-range PCR protocol. Our raven mitogenomes ranged between 16925–16928 bp in length. GC content varied from 43.3% to 43.8% and the 13 protein coding genes, two rRNAs and 22 tRNAs followed a standard avian mitochondrial arrangement. The overall divergence between the two common raven clades was 3% (range 0.3–5.8% in 16 regions including the protein coding genes, rRNAs and the control region). Phylogenies constructed from whole mitogenomes recovered the previously found mitochondrial sister relationship between the common raven California clade and the Chihuahuan raven (overall divergence 1.1%), which strengthens the hypothesis that mtDNA paraphyly in the common raven results from speciation reversal of previously distinct Holarctic and California lineages.

## Introduction

In the era of genomics and high throughput sequencing, complete mitochondrial genomes (mitogenomes) are rapidly becoming available for many organisms [1]. In addition to increasing our knowledge of functional aspects of mitochondrial genes and molecular evolution [2, 3], complete mitogenomes offer increased resolution in phylogenetic and phylogeographic analyses [4–9]. Furthermore, obtaining complete mitogenomes reduces the likelihood of sequencing nuclear pseudogenes instead of mitochondrial DNA [10].

The number of published avian mitogenomes is increasing, and to date such genomes are available for more than 1530 specimens from more than 640 species in GenBank. Within the genus *Corvus*, there are complete mitogenomes available for seven species, but none from the clade of large-bodied raven species (clade V in [11]) distributed in the Afro-Holarctic region (Africa: 6 species; south-west North America: 1 species; Holarctic: 1 species). While the monophyly of the Afro-Holarctic raven clade is well established, relationships within the clade remain uncertain—especially those within and between the *Corvus ruficollis* species group (a complex of four morphologically variable crow-sized species in Africa: *C*. *ruficollis*, *C*. *edithae*, *C*. *albus*, *C*. *rhipidurus*), and the common raven species group (a complex of two larger-bodied species distributed across the Northern Hemisphere: the common raven *C*. *corax*, and Chihuahuan raven *C*. *cryptoleucus*) [11–14]. Here we focus on the common raven species group.

The common raven (*Corvus corax* L.) is one of the world’s largest passerines, with a wide breeding distribution in the Northern Hemisphere. Previous studies based on several mitochondrial markers (control region, cytochrome *b*, cytochrome *c* oxidase subunit 1 (COI)) show that this species harbours two highly diverged mtDNA lineages [15–17]. Structuring between California and Holarctic lineages is also apparent in microsatellite data [17], but not in the single nuclear intron studied to date (beta-fibrinogen intron,[12]). The Holarctic lineage is found across Eurasia and North America, while the California lineage is restricted to western North America where it co-occurs with the Holarctic lineage [15, 17]. Despite this deep divergence, there is no evidence that the two lineages are reproductively isolated as field studies suggest that they interbreed to a large extent and their offspring are viable [18]. Common ravens are therefore hypothesised to be an example of speciation reversal of divergent lineages upon secondary contact [18]. A further complication to the apparently reticulate history of the common raven is the fact that a second sympatric species, the Chihuahuan raven (*Corvus cryptoleucus*) restricted to south-western North America, is nested within the common raven based on mitochondrial DNA, having a closer relationship to the Californian clade, than either of them have to the Holarctic clade [12, 17]. However, these two species are phenotypically distinct, and there is no evidence of hybridization from either field or mtDNA studies [12, 17–19]. Overall, there appears to be good support for reproductive isolation between common and Chihuahuan ravens despite mtDNA paraphyly.

In this study, we present the first mitogenomes for Afro-Holarctic ravens. We sequence three raven species—the common raven, the Chihuahuan raven and the brown-necked raven (*Corvus ruficollis*)—and test whether mtDNA paraphyly results from reticulations in the speciation history of ravens. If complete mitogenomes and all constituent gene regions recover the deeply divergent California and Holarctic mtDNA clades and support paraphyly of common ravens with respect to Chihuahuan ravens, this offers evidence that paraphyly originates from a reticulate speciation history—most likely one where the mtDNA tree reflects an earlier divergence between the Holarctic lineage and the ancestor of California and Chihuahuan lineages followed by a more recent divergence of California and Chihuahua lineages. Alternatively, if complete mtDNA genomes reconstruct a different topology to that recovered in previous phylogenetic analyses of single mtDNA loci [15–17], this could suggest that apparent mtDNA paraphyly of common ravens resulting from a close sister relationship of California clade and Chihuahuan ravens could be caused by previous studies inadvertently sequencing nuclear pseudogenes.

## Materials and methods

**Fig 1:**
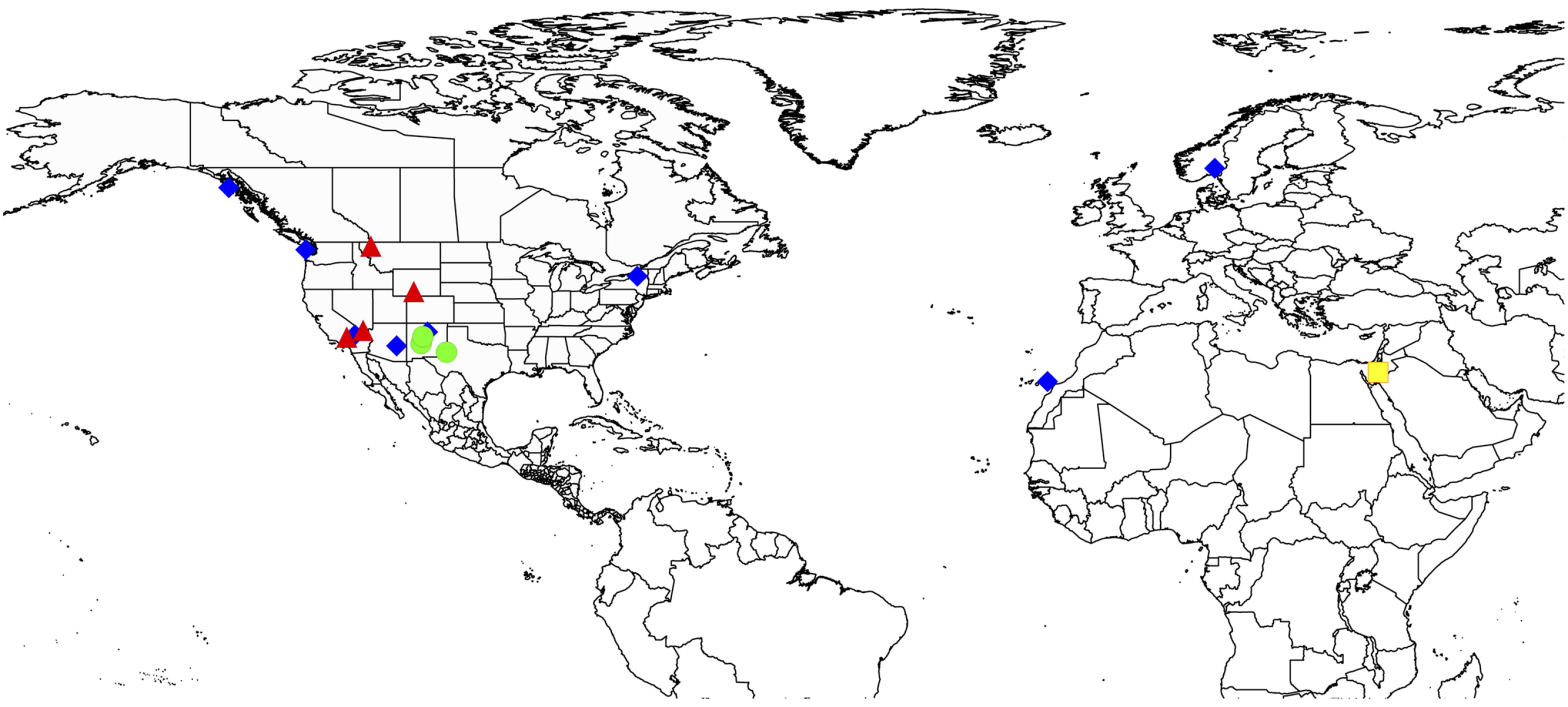
Map showing sampling locations. Blue diamond = *C*. *corax*, Holarctic lineage; red triangle = *C*. *corax*, California lineage; green circle = *C*. *cryptoleucus*; yellow square = *C*. *ruficollis*.

Blood samples were obtained from live birds (n = 8) and tissue samples from frozen tissue collections at official US natural history museums (n = 8). Seven of eight live-sampled birds were adults caught in mist nets/rocket nets, while the last one was a chick sampled in the nest. All birds were released in good condition immediately after blood sampling. All samples had been collected with the appropriate banding and collecting permits from relevant national authorities. Table 1 summarises specimen details and Fig 1 shows the geographic position of sampling locations. We analysed 12 samples of *Corvus corax* (10 from North America, 1 from Norway and 1 from the distinct Canary islands lineage that is nested within the Holarctic lineage [20]), three *Corvus cryptoleucus* (from New Mexico in south-west USA) and one *Corvus ruficollis* (from Israel). Of the 10 *Corvus corax* from North America, two were from northern and eastern regions with pure Holarctic mtDNA (Alaska, New York), two were from the state of California where the majority of ravens have California mtDNA, and six were from within the region of overlap of Holarctic and California mtDNA lineages in western North America (Arizona, New Mexico, Nevada, Washington, Wyoming, Montana) [18]. See Table 1 for mtDNA clade assignments of each sample based on single mtDNA loci from previous studies ([17, 18]; authors' unpubl. data).

**Table 1:** Sample information. Clade assignment of common ravens from North America to either California or Holarctic lineages are given for mitogenomes presented in this study and either mtDNA control region or COI sequenced in previous studies ([17, 18]; authors’ unpubl. data).

Species	Accession number	Sampling locality	Coordinates (decimal degrees)	Collection date	Clade assignment	
					Mito-genome	Single mtDNA locus
*C*. *corax*	2407–51899	San Bernardino, CA, USA	35.26N, 116.68W	22.05.2001	HOL	HOL
*C*. *corax*	USFWS 2327–69957	Gila, AZ, USA	33.64N, 110.52W	25.04.2005	HOL	HOL
*C*. *corax*	UAM30328	Sitka, AK, USA	57.05N, 135.33W	2012	HOL	HOL
*C*. *corax*	NYSM 11227	Hamilton, NY, USA	43.95N, 74.94W	13.08.2011	HOL	HOL
*C*. *corax*	MSB21677	Santa Fe, NM, USA	35.69N, 105.94W	2009	HOL	HOL
*C*. *corax*	2387–36563	Jefferson, WA, USA	47.86N, 123.94W	17.05.1997	HOL	HOL
*C*. *corax*	NHMO-BI-23199	Maridalen, Oslo, Norway	60.00N, 10.79E	04.05.2010	HOL	HOL
*C*. *corax*	NHMO-BI-35585	Fuerteventura, Spain	28.35N, 14.03W	2013	HOL	HOL
*C*. *corax*	1547–43719	Wamsutter, WY, USA	41.67N, 107.98W	2013	CAL	CAL
*C*. *corax*	UCSB 90–175	Kern, CA, USA	34.92N, 117.89W	07.12.1994	CAL	CAL
*C*. *corax*	1807–88239	Flathead, MT, USA	48.39N, 114.33W	23.04.2014	CAL	CAL
*C*. *corax*	MBM 9200	Clark, NV, USA	35.93N, 115.47W	18.05.2005	CAL	CAL
*C*. *cryptoleucus*	MSB25417	Socorro, NM, USA	33.99N, 106.89W	2005	na	na
*C*. *cryptoleucus*	MSB40523	Bernalillo, NM, USA	35.02N, 106.63W	2013	na	na
*C*. *cryptoleucus*	MSB22405	Lea, NM, USA	32.70N, 103.14W	1999	na	na
*C*. *ruficollis*	NHMO-BI-18431	Eilat, Israel	29.55N, 34.93E	21.03.2009	na	na

### Molecular analyses

DNA was extracted with EZNA blood/tissue kits (Omega Inc), following the protocol of the manufacturer. For sequencing of the complete mitogenomes, we followed the protocol recently described by Lifjeld *et al*. [21]. Briefly, mitochondrial DNA was amplified from high molecular weight genomic DNA using two primer pairs: mtCorvus531F (GGATTAGATACCCCACTATGC) & mtCorvus9431R (GTCTACRAAGTGTCAGTATCA) and mtCorvus8031F (CCTGAWCCTGACCATGAACCTA) & mtCorvus926R (GAGGGTGACGGGCGGTATGTA). These two primer pairs yielded respective amplicons with ~8,900 bp and ~9,800 bp. The primers were designed to anneal in conserved regions of the mitogenome, based on an alignment with genetic information from the following species: *Corvus frugilegus*, *Corvus splendens*, *Corvus corax*, *Taeniopygia guttata*, *Molohthrus aeneus*, *Ficedula albicollis*, *Melamprosops phaeosoma* and *Gallus gallus*. Annealing sites and overlapping regions are illustrated in Lifjeld *et al*. [21]. The following PCR conditions were utilized for amplification: 1X reaction buffer, 200 μM of each dNTP, 0.5 μM of each primer, ~20 ng template DNA, 0.02 U/μl Q5 High-Fidelity DNA polymerase (New England Biolabs) and dH2O to a final volume of 25 μl. The following thermal profiles were employed: Amplicon 1 –Initial denaturation 98°C in 30 seconds, 35 cycles with denaturation 98°C for 10 seconds, annealing 59°C for 20 seconds and elongation 72°C for 7.5 minutes, and a final elongation step for 2 minutes. Amplicon 2 –Initial denaturation 98°C in 30 seconds, 5 cycles with denaturation 98°C for 10 seconds following a touch down profile starting at 72°C with 1°C/cycle reduction, 30 cycles with denaturation 98°C for 10 seconds, annealing 67°C for 20 seconds and elongation 72°C for 7.5 minutes, and a final elongation step for 2 minutes.

The complete PCR reactions were transferred to a 0.8% agarose gel and ran at 90V. When completely separated, the respective amplicons were cut from the gel and purified using the GenJet Gel Extraction Kit (ThermoFischer Scientific). Concentrations of the purified amplicons were measured on a Qubit instrument (ThermoFischer Scientific) and equimolar amounts of each amplicon were pooled. Approximately 20 ng of pooled amplicons from each individual where sheared using a Covaris M220 Focused-ultrasonicator (Covaris, Inc.), running the pre-programmed DNA shearing protocol for 800 bp twice. Size selection was performed using a BluePippin (Sage Science) instrument. We size selected DNA in the 350–450 bp range using a 2% agarose gel cassette (Sage Science). To generate barcoded libraries for sequencing, we employed the NEBNext library prep kit for Ion Torrent (New England Biolabs) on the sheared size selected amplicons using the IonXpress barcode adapter kit (ThermoFischer Scientific). Barcoded libraries were pooled and concentration of the final library was measured on a Fragment Analyzer (Advanced Analytical) using the DNF-474 High Sensitivity NGS Fragment Analysis kit. The size selected, barcoded, sheared amplicons were sequenced on a 314 chip using an IonPGM sequencing instrument (ThermoFischer Scientific).

### Bioinformatics

Trimming, removal of low quality reads and demultiplexing were performed on the Torrent Suite^™^ software (ThermoFisher Scientific). The *Corvus splendens* mitogenome (GenBank acc. NC024607; [22]) was used as reference in the Torrent Suite ^™^ software for coverage estimates, using the plugin coverageAnalysis (v4.4.2.2). Additional trimming to improve quality of the data was performed using Trimmomatic v0.33 [23] with the following settings: Sliding window 4:20. Minimum read length:100. Mitogenomes were reconstructed by iterative mapping using MITObim v1.8 [24]. The complete mitogenome of *Corvus splendens* was used as reference in the mapping.

Duplications in the region between the *cytB* gene and the *12S rRNA* gene have frequently been observed among bird taxa (e.g. [4, 25]) and such duplications may be indicated by increased coverage in this region [26]. We addressed this issue by inspecting coverage plots, emphasizing the area where duplications are likely to occur. This was performed in the software Tablet v1.14.10.20 [27]. We observed a drop in coverage in the start of the complement strand of the *NAD6* gene. Hence, we employed a second iterative mapping using the assembled *NAD6* gene as short sequence bait. Using this strategy, we avoided having a reference that covers the complete problematic region where duplications have been shown to occur. If the reference sequence covers this area, one may expect a bias in assembled reads based on similarity to the reference. In other words, if the true sequence includes a duplicated region and the reference sequence does not, the duplication may be masked since only the most similar reads will map to the reference. When using a short sequence bait, the assembly will be generated from the iterative mapping and accordingly independently from a large reference sequence. We found that the region where the coverage dropped had a local GC content of 72% and contained two stretches of C_(n)_ homopolymers, which have been shown to introduce errors in sequence data generated by the Ion Torrent platform [28]. Such local high GC content may lead to systematic coverage drop in vertebrate mitochondria assemblies [29]. This may result from DNA polymerase introducing premature elongation stops in the GC rich region during the library amplification or from incomplete nucleotide incorporation in the sequencing reaction. We observed a major overrepresentation of reverse reads in the region with the coverage drop, indicating incomplete sequencing of forward reads through the respective region. We also observed a similar coverage drop in the same region in Illumina generated data [30]. Furthermore, our contig sequences were identical to the first iterative mapping when a short *NAD6* sequence was used as bait. Hence, we conclude that the observed drop in coverage in our dataset is likely explained by the biochemical properties of the template DNA and/or the chemical reagents and not because of gene order alterations.

We were not able to cover the complete control region in all individuals in our assemblies, but for both of the common raven clades and the Chihuahuan raven we obtained the complete control region from at least one individual. For the single individual of brown-necked raven, we lack ~140 bp from the end of the control region, and the total mitogenome length for this individual was estimated based on the alignment of the other study specimens.

Mitochondrial genes were first automatically annotated using MITOs [31], and thereafter manually inspected.

### Phylogenetic analyses

Genetic distances for nucleotides were measured as the number of base substitutions divided by the respective sequence length, averaging over all sequence pairs between groups (uncorrected p-distance). Standard error estimates were obtained by a bootstrap procedure (100 replicates). Genetic distance analyses were conducted using the maximum composite likelihood model [32]. The coding data was translated assuming a vertebrate mitochondrial genetic code. Genetic distance estimates were calculated in MEGA7 [33].

Given that accumulation of substitutions may bias phylogenetic interpretations, we performed substitution saturation tests on five different data sets: (i) all codon positions in protein coding genes (11,381 bp), (ii) first and second codon position in all protein coding genes (7,586 bp), (iii) first codon position (3,793 bp), (iv) second codon position and (v) third codon position. We followed Xia & Lemey [34] to estimate the index of substitution saturation (I_SS_). The I_SS_ statistics were performed on the gap-free sites in the specified data sets using the software DAMBE 6.3.17 [34, 35]. The I_SS_ values were significantly lower (p <0.0001) than the critical I_SS_ values for all the data sets (S1 Table), indicating no saturation of substitutions. Accordingly we included all codon positions and non-coding nucleotide position in our phylogenetic analyses.

ClustalW was used to align our raven mitogenome dataset with previously published mitogenomes available for seven other species of *Corvus*–*C*. *brachyrhynchos*, *C*. *cornix*, *C*. *frugilegus*, *C*. *hawaiiensis*, *C*. *macrorhynchos*, *C*. *moriorum*, and *C*. *splendens*–and *Pica pica*, which were obtained from GenBank (accession numbers appear in the figures). The alignment was manually inspected in MEGA7. Maximum likelihood (ML) trees were constructed in MEGA7 using *Pica pica* as an outgroup. ML trees used the best fit substitution model according to Bayesian information criterion and Akaike information criterion (general time reversible + G + I) as estimated by MEGA7. Initial tree for the heuristic search was obtained by applying the maximum parsimony method [36]. The tree with the highest log likelihood (-52,715) was chosen for visualization. Test of node support in the phylogenetic tree was performed using 1,000 bootstrap replicates [37]. All positions with less than 95% coverage in the alignment were eliminated. ML trees were also constructed from nucleotide subsets containing the minimum (*16S rRNA*) and the maximum (*ATP6*) genetic distance between the clades (S1 and S2 Figs), and from subsets containing only protein coding genes (S3 Fig) and the control region (S4 Fig), which has been the region used most extensively to study the geographic distribution of Holarctic and California clades in western North America [18, 38].

## Results

The Ion PGM run yielded 1,022,295 reads. In total, 45.2% was filtered as polyclonals and 9.3% as low quality reads in the Torrent Suite software. Sample specific information regarding total number of reads, trimmed reads, mapped reads, coverage and GenBank accessions numbers are provided in S2 Table.

**Fig 2:**
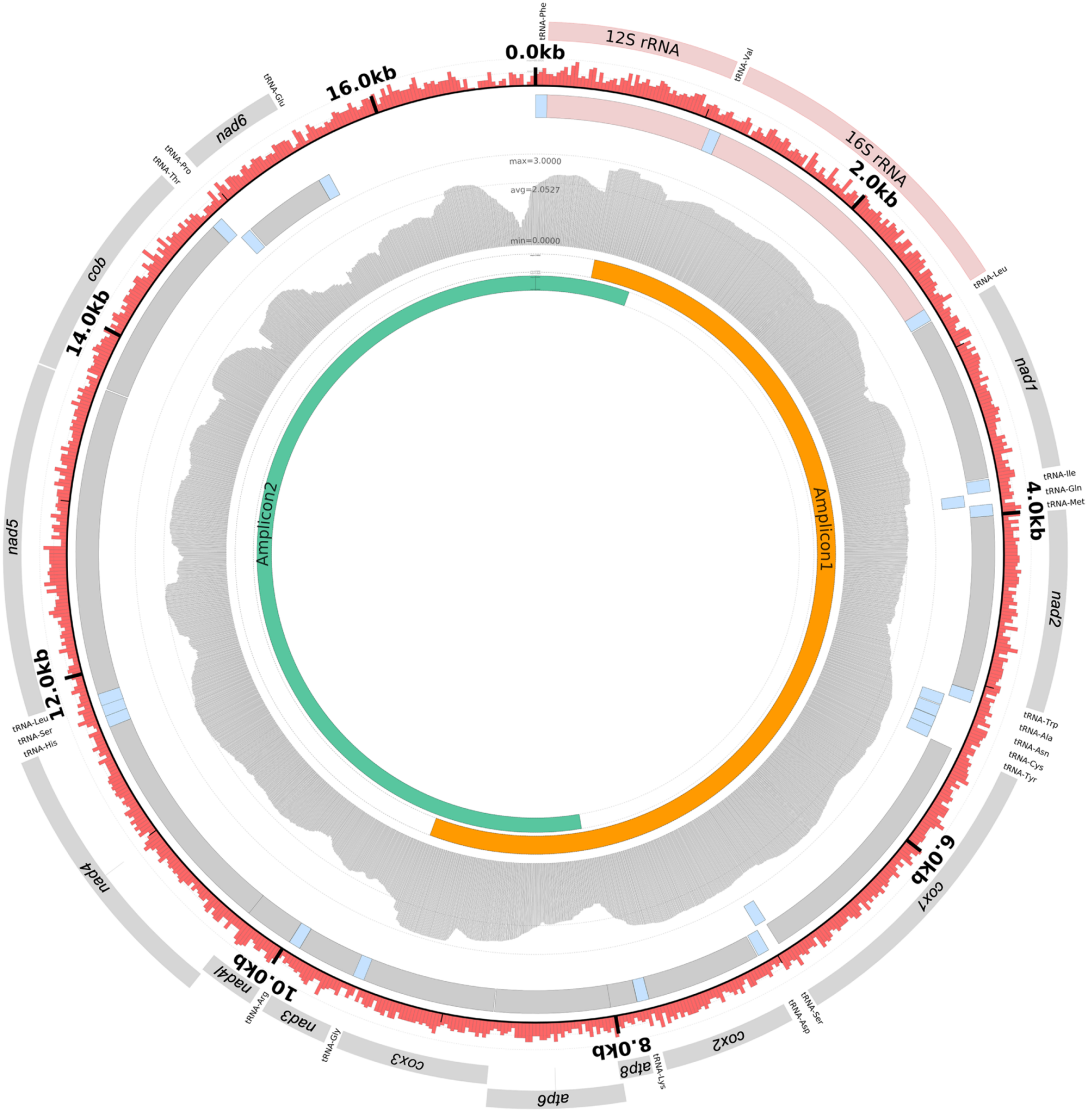
Graphical overview of the *Corvus corax* mitogenome. The figure contains the following information, from the outermost to the innermost layer: (1) Gene products for annotated genes. (2) Genome position, minor ticks for every kb. Red bars illustrate GC-content in 20 bp windows. (3) Forward and (4) reverse genes. Blue colours show tRNAs, red colours show rRNAs and grey colours illustrate protein coding genes. (5) Coverage plot with log transformed coverage. (6) Position for amplicon 1 and (7) position for amplicon 2. The figure was created from GenBank accession KX245135 in the software Circleator [42].

The total length of the raven mitogenomes ranged between 16,925 bp (Californian clade) and 16,928 bp (Holarctic clade), while that of the Chihuahuan raven was 16,928 bp and the brown-necked raven 16,927 bp. Gene annotation analyses revealed 13 protein coding genes, 2 rRNAs and 22 tRNAs (Fig 2). The gene arrangement followed a standard avian mitogenome model [39], similar to published mitogenomes from other *Corvus* species [22, 40, 41]. Analyses of base composition revealed a GC content of 43.8% (Holarctic clade), 43.6% (Californian clade), 43.7% (Chihuahuan raven) and 43.3% (brown-necked raven).

**Fig 3:**
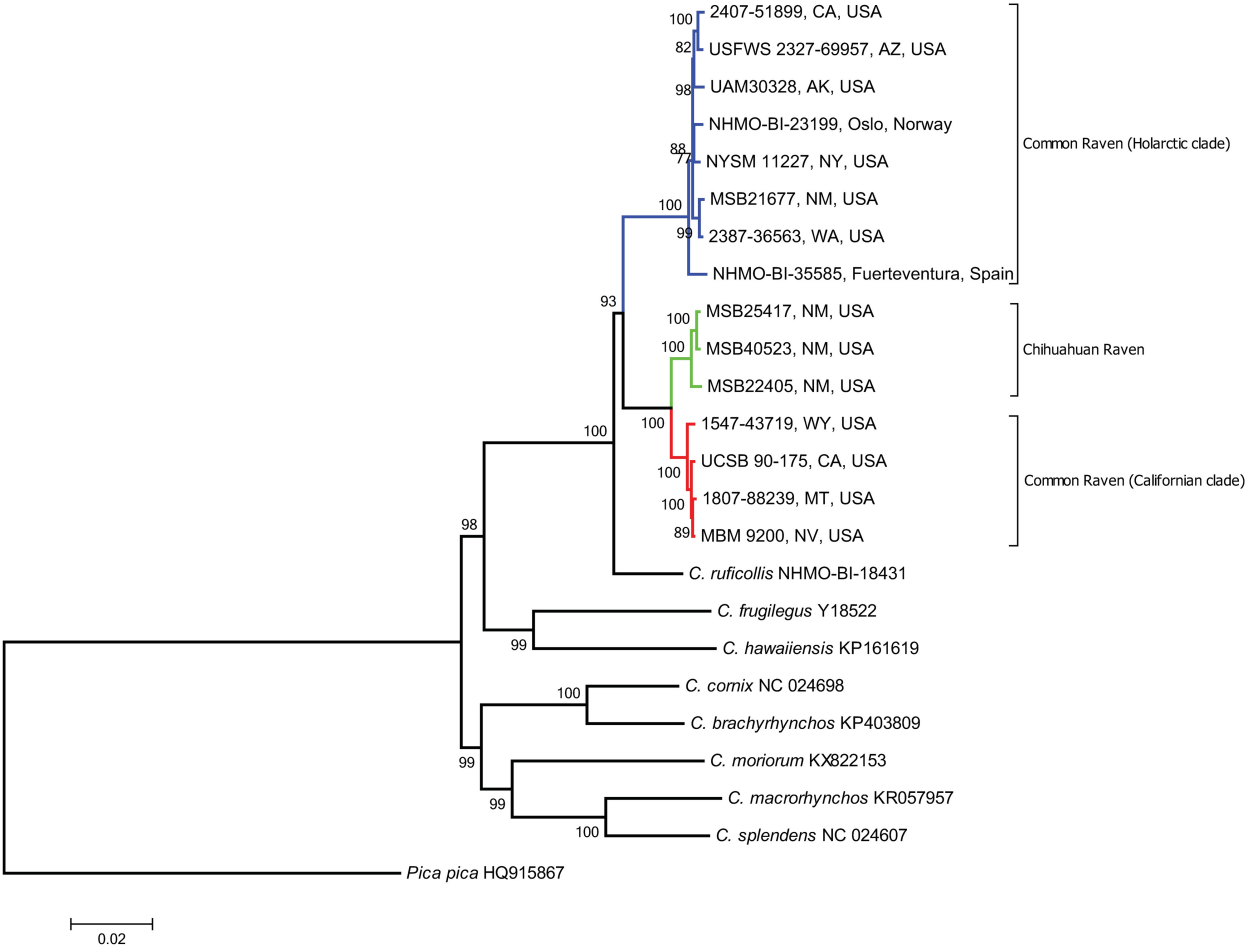
Maximum likelihood phylogeny inferred from whole mitogenomes of three species of ravens (common raven *C*. *corax*, Chihuahuan raven *C*. *cryptoleucus*, brown-necked raven *C*. *ruficollis*), and six other *Corvus* species with published mitogenomes (labeled with GenBank acc. no.). The final dataset used in the phylogenetic analysis contained 15,392 bp after indels and positions in the alignment with less than 95% coverage were eliminated. The tree was rooted with *Pica pica*. USA = United States of America, CA = California, AZ = Arizona, AK = Alaska, NY = New York, NM = New Mexico, WA = Washington, WY = Wyoming, MT = Montana, NV = Nevada.

There was full agreement between the clade assignment and phylogenetic relationships based on previous single-marker mtDNA studies ([17, 18]; authors' unpubl. data) and that based on the complete mitogenome (Fig 3; Table 1). The overall sequence divergence across the alignment between birds belonging to the Holarctic and the Californian clade of common ravens was 3%. However, there was a large degree of variation across the 13 protein coding genes, the two rRNA genes and the control region, ranging from 0.3% in *16S rRNA* to 5.8% in *ATP6* (Table 2). The divergence between the Holarctic clade and the Chihuahuan raven was very similar to that between the Holarctic and the Californian Clade (overall: 3.1%; range across regions: 0.3% - 5.5%; Table 2), while the Californian clade and the Chihuahuan raven was on average around two percentage points less diverged (overall: 1.1%; range across regions: 0.2% - 2.8%; Table 2).

**Table 2:** Genetic distances (uncorrected p-distances) among Holarctic (HOL) and Californian (CAL) lineages of the common raven and the Chihuahuan (CHI) raven, for different partitions, the whole alignment and protein coding genes concatenated.

Gene/Region	Comparison	Distance	SE	Length (bp)^a^
*12S rRNA*	HOL-CAL	0.0046	0.0022	980
*12S rRNA*	HOL-CHI	0.0045	0.0021	980
*12S rRNA*	CAL-CHI	0.0020	0.0014	980
*16S rRNA*	HOL-CAL	0.0027	0.0013	1,601
*16S rRNA*	HOL-CHI	0.0023	0.0013	1,601
*16S rRNA*	CAL-CHI	0.0025	0.0013	1,601
*NAD1*	HOL-CAL	0.026	0.008	975
*NAD1*	HOL-CHI	0.026	0.008	975
*NAD1*	CAL-CHI	0.007	0.003	975
*NAD2*	HOL-CAL	0.031	0.005	1,041
*NAD2*	HOL-CHI	0.032	0.006	1,041
*NAD2*	CAL-CHI	0.021	0.004	1,041
*COXI*	HOL-CAL	0.026	0.004	1,551
*COXI*	HOL-CHI	0.026	0.004	1,551
*COXI*	CAL-CHI	0.004	0.002	1,551
*COXII*	HOL-CAL	0.019	0.005	681
*COXII*	HOL-CHI	0.020	0.005	681
*COXII*	CAL-CHI	0.011	0.003	681
*ATP8*	HOL-CAL	0.033	0.013	153
*ATP8*	HOL-CHI	0.023	0.012	153
*ATP8*	CAL-CHI	0.010	0.004	153
*ATP6*	HOL-CAL	0.058	0.009	684
*ATP6*	HOL-CHI	0.055	0.009	684
*ATP6*	CAL-CHI	0.028	0.007	684
*COXIII*	HOL-CAL	0.023	0.005	738
*COXIII*	HOL-CHI	0.025	0.005	738
*COXIII*	CAL-CHI	0.008	0.003	738
*NAD3*	HOL-CAL	0.036	0.010	348
*NAD3*	HOL-CHI	0.038	0.010	348
*NAD3*	CAL-CHI	0.009	0.005	348
*NAD4l*	HOL-CAL	0.048	0.012	290
*NAD4l*	HOL-CHI	0.046	0.012	290
*NAD4l*	CAL-CHI	0.012	0.005	290
*NAD4*	HOL-CAL	0.034	0.008	1,377
*NAD4*	HOL-CHI	0.031	0.007	1,377
*NAD4*	CAL-CHI	0.011	0.003	1,377
*NAD5*	HOL-CAL	0.046	0.006	1,815
*NAD5*	HOL-CHI	0.048	0.006	1,815
*NAD5*	CAL-CHI	0.013	0.002	1,815
*COB*	HOL-CAL	0.039	0.007	1,093
*COB*	HOL-CHI	0.045	0.007	1,093
*COB*	CAL-CHI	0.017	0.004	1,093
*NAD6*	HOL-CAL	0.043	0.010	518
*NAD6*	HOL-CHI	0.042	0.010	518
*NAD6*	CAL-CHI	0.013	0.005	518
*CR*	HOL-CAL	0.039	0.005	1,198
*CR*	HOL-CHI	0.043	0.005	1,198
*CR*	CAL-CHI	0.022	0.004	1,198
Whole mt-genome	HOL-CAL	0.029	0.001	16,553
Whole mt-genome	HOL-CHI	0.030	0.001	16,553
Whole mt-genome	CAL-CHI	0.011	0.001	16,553
Protein coding genes	HOL-CAL	0.037	0.002	11,275
Protein coding genes	HOL-CHI	0.038	0.002	11,275
Protein coding genes	CAL-CHI	0.013	0.001	11,275

^a^ All alignment positions with missing data (gaps or ambiguous bases) were eliminated.

The monophyly of the Afro-Holarctic raven clade was also supported (bootstrap support = 100; Fig 3), as per previous studies based on single mtDNA loci or concatenation of nuclear introns and mtDNA [11–14]. The maximum likelihood tree estimated from whole mitogenomes strongly supported a sister relationship between the Californian clade of the common raven and the Chihuahuan raven (bootstrap support = 100; Fig 3). There was strong support for a sister relationship between the Holarctic clade and the clade containing both the Chihuahuan raven and the California clade (bootstrap support = 93; Fig 3). ML trees constructed from four different subsets of the mitogenome dataset (*16S rRNA*, *ATP6*, all protein coding genes, control region; S1–S4 Figs) recovered similar topologies as the whole mitogenome tree (Fig 3). As expected *16S rRNA* showed the least resolution with generally low bootstrap values. For the other three subsets, we found high support for the monophyly of the Afro-Holarctic raven clade (98–100) and each of its three multi-individual terminal clades (Holarctic clade, California clade, Chihuahuan raven; 97–100), as well as for the sister relationship between the California clade and the Chihuahuan raven (94–100). The single brown-necked raven was placed outside these three clades in the *ATP6* and all protein coding genes subsets, with moderate support (72–79), but came out together with the Holarctic clade in the control region tree, again with moderate support (75). Uncertainty with respect to the monophyly of the common raven species group in the different subsets could be explained by ambiguity stemming from variable levels of divergence with respect to the brown-necked raven.

## Discussion

Whole mitogenome sequences confirmed previous findings based on single mitochondrial markers; a deep split between Holarctic and California clades within the common raven and a sister relationship between the California clade and the Chihuahuan raven (Fig 3) [12, 17]. The deep split was present in all parts of the mitogenome, although the different regions showed different levels of divergence (S1–S4 Figs), reflecting variable mutation rates among mitochondrial regions [43]. The sister relationship between the Californian clade and the Chihuahuan raven was also confirmed in all but the least diverged region (*16S rRNA*; S1 Fig). The apparent conflict between species boundaries supported by phenotypic and behavioural traits and the mtDNA tree can be explained by the speciation reversal hypothesis, wherein the mtDNA tree reflects the “original” divergence history of three previously distinct raven lineages (Holarctic, Californian and Chihuahuan) before secondary contact and speciation reversal of the Holarctic and California lineages into a single admixed species, the common raven [18]. An alternative hypothesis is that the mtDNA sister relationship of California and Chihuahuan ravens results from ancient introgressive hybridization between the two, which resulted in the capture and replacement of one species’ original mitochondrial lineage with the others’ (see [44] for a similar case in *Emberiza* buntings), with subsequent divergence. Differentiating between these three hypotheses—(1) mtDNA phylogeny reflects the original speciation history prior to speciation reversal of California and Holarctic lineages, (2) Chihuahuan ravens have mtDNA captured from ancient introgressive hybridization with California clade common ravens, (3) California clade common ravens have mtDNA captured from ancient introgressive hybridization with Chihuahuan ravens—would require the addition of nuclear genomic data and is beyond the scope of this study. However, the consistent signal of divergence and paraphyly combined with a lack of stop codons and frameshift mutations throughout the mitogenome, strongly supports a true mitochondrial origin of the highly diverged raven lineages.

Our study illustrates the advantages of using whole mitogenomes to resolve phylogenetic relationships. The high resolution of our whole mitogenome phylogeny suggests that uncertainty about relationships between the eight species of Afro-Holarctic ravens could be resolved with additional sequencing of mitogenomes from the species missing from our mitogenome dataset—i.e., *C*. *albus*, *C*. *edithae*, *C*. *rhipidurus*, *C*. *albicollis*, and *C*. *crassirostris*. Inclusion of the remaining members of the *C*. *ruficollis* species group is particularly critical given that some studies have supported these species as closer to the Holarctic common raven clade than the Holarctic clade is to either the California common raven clade and the Chihuahuan raven (i.e., paraphyly of the “common raven species group”; [12, 13]). This conflicts with our strong support for the monophyly of the “common raven species group” to the exclusion of *C*. *ruficollis* in the analysis based on whole mitogenomes (Fig 3). Note, however, that a sister relationship between *C*. *ruficollis* and the Holarctic clade was recovered in the control region (S4 Fig), albeit with rather weak support. Given the conflict between phylogenies based on mitogenomes (this study), single loci [13] and multilocus datasets [11, 12, 45], and considering reports that many of the species in the *C*. *ruficollis* species group hybridize with each other [19], we suggest that future attempts to produce a fully resolved phylogeny for the Afro-Holarctic ravens should include multiple individuals from the ranges of all species (including regions of allopatry and sympatry where possible), incorporate whole mitogenomes, and extensive sampling of the nuclear genome (e.g. sequence capture or whole genome re-sequencing approaches).

In conclusion, mitogenomes support previously found deep lineages and paraphyly in the common raven/Chihuahuan raven species complex. Our sequencing strategy provides 16 new mitogenomes with high coverage even from the smallest IonPGM chip, showing that our approach of long-range PCR amplification and IonPGM sequencing provides a cost efficient alternative to mitogenome assembly from whole genome sequencing data.

## Supporting information

S1 Fig Maximum likelihood trees of 16S rRNA (1,599 positions). The tree was rooted with *Pica pica*. USA = United States of America, CA = California, AZ = Arizona, AK = Alaska, NY = New York, NM = New Mexico, WA = Washington, WY = Wyoming, MT = Montana, NV = Nevada.
(TIF)

S2 Fig Maximum likelihood trees of ATP6 (681 positions). The tree was rooted with *Pica pica*. USA = United States of America, CA = California, AZ = Arizona, AK = Alaska, NY = New York, NM = New Mexico, WA = Washington, WY = Wyoming, MT = Montana, NV = Nevada.
(TIF)

S3 Fig Maximum likelihood trees of protein coding genes (11,186 positions). The tree was rooted with *Pica pica*. USA = United States of America, CA = California, AZ = Arizona, AK = Alaska, NY = New York, NM = New Mexico, WA = Washington, WY = Wyoming, MT = Montana, NV = Nevada.
(TIF)

S4 Fig Maximum likelihood trees of control region (1,166 positions). *C moriorum* was excluded from the tree due to missing data. The tree was rooted with *Pica pica*. USA = United States of America, CA = California, AZ = Arizona, AK = Alaska, NY = New York, NM = New Mexico, WA = Washington, WY = Wyoming, MT = Montana, NV = Nevada.
(TIF)

S1 Table Test of mutational saturation in the five data sets with the 13 mitochondrial protein coding genes. The table provide index of substitution saturation (I_SS_) and critical values of I_SS_ for symmetric and asymmetric tree topologies. The I_SS_ values were significantly lower (p <0.0001) than the critical I_SS_ in all data sets.
(DOCX)

S2 Table Accession number, total number of reads, number of reads after trimming, reads assembled in the iterative mapping, average coverage in MITObim assembly, GenBank accession numbers and GenBank BioSample ID for each sample. (DOCX)
