# Supplementary figures and images for: Sequencing of the complete mitochondrial genome of the common raven Corvus corax (Aves: Corvidae) confirms mitogenome-wide deep lineages and a paraphyletic relationship with the Chihuahuan raven C. cryptoleucus
Source: PLoS One. 2017 Oct 30;12(10):e0187316. doi: 10.1371/journal.pone.0187316 (PMC5662180; doi:10.1371/journal.pone.0187316)

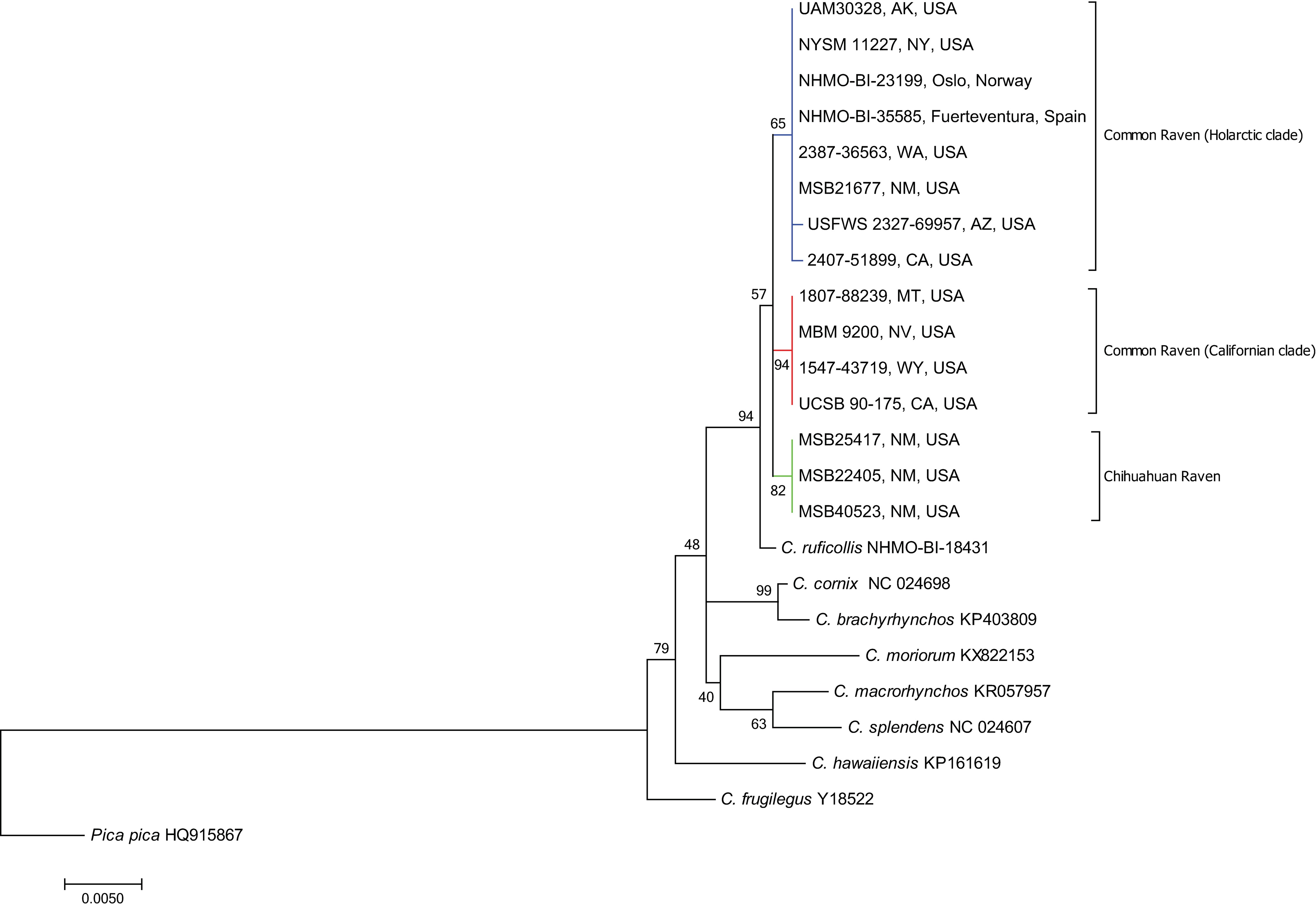

Supplement: S1 Fig — The tree was rooted with Pica pica. USA = United States of America, CA = California, AZ = Arizona, AK = Alaska, NY = New York, NM = New Mexico, WA = Washington, WY = Wyoming, MT = Montana, NV = Nevada. (TIF) [file pone.0187316.s001.tif]

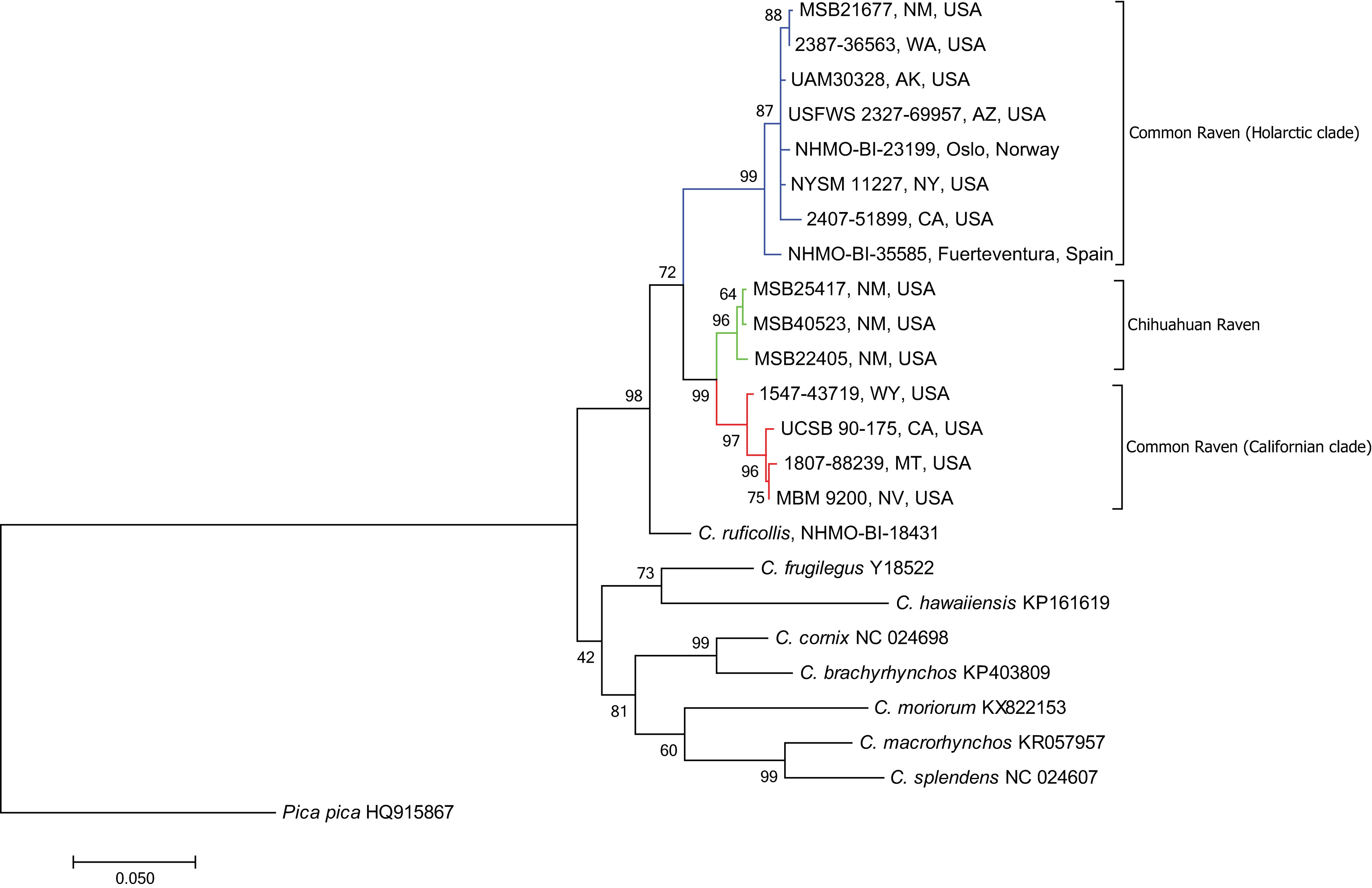

Supplement: S2 Fig — The tree was rooted with Pica pica. USA = United States of America, CA = California, AZ = Arizona, AK = Alaska, NY = New York, NM = New Mexico, WA = Washington, WY = Wyoming, MT = Montana, NV = Nevada. (TIF) [file pone.0187316.s002.tif]

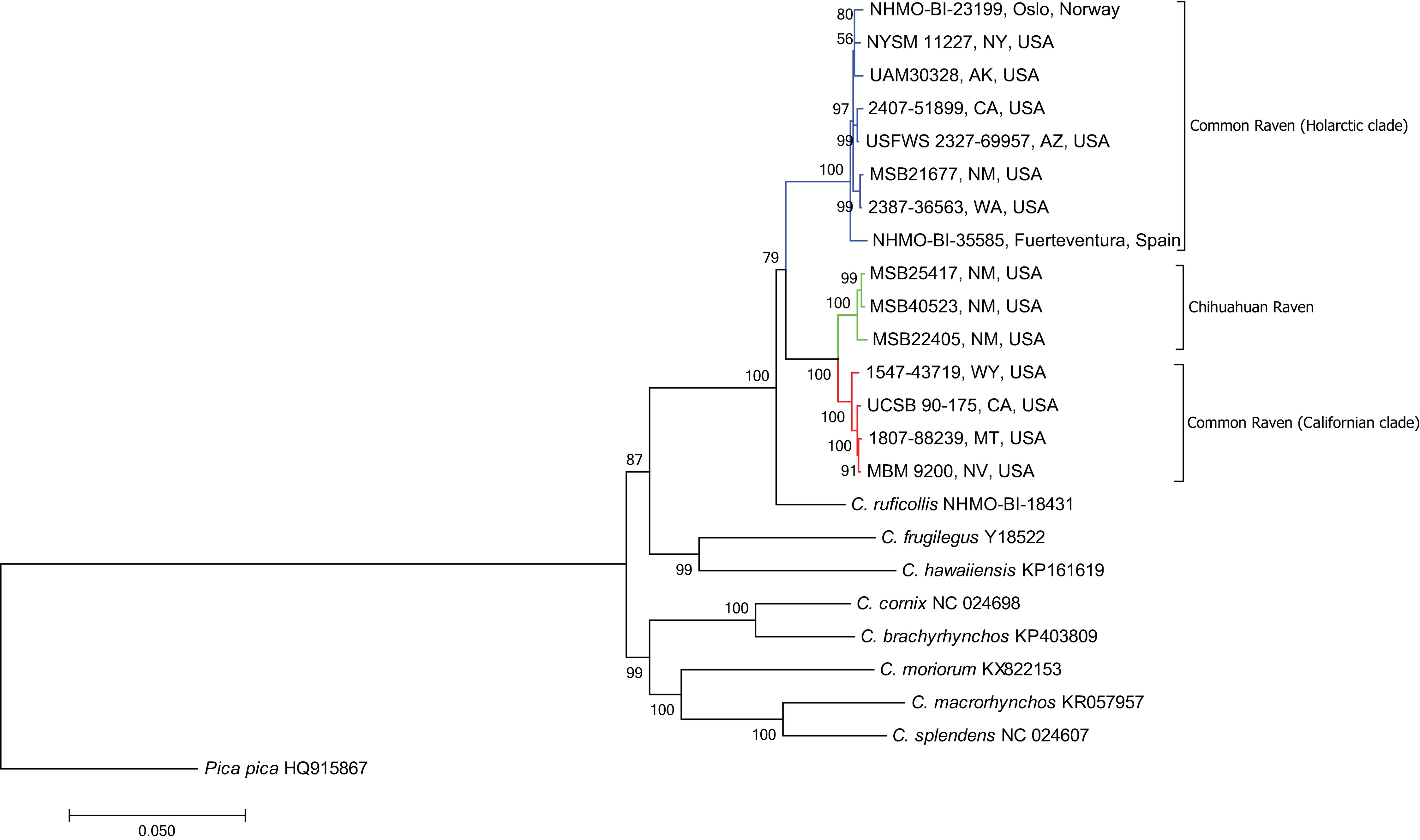

Supplement: S3 Fig — The tree was rooted with Pica pica. USA = United States of America, CA = California, AZ = Arizona, AK = Alaska, NY = New York, NM = New Mexico, WA = Washington, WY = Wyoming, MT = Montana, NV = Nevada. (TIF) [file pone.0187316.s003.tif]

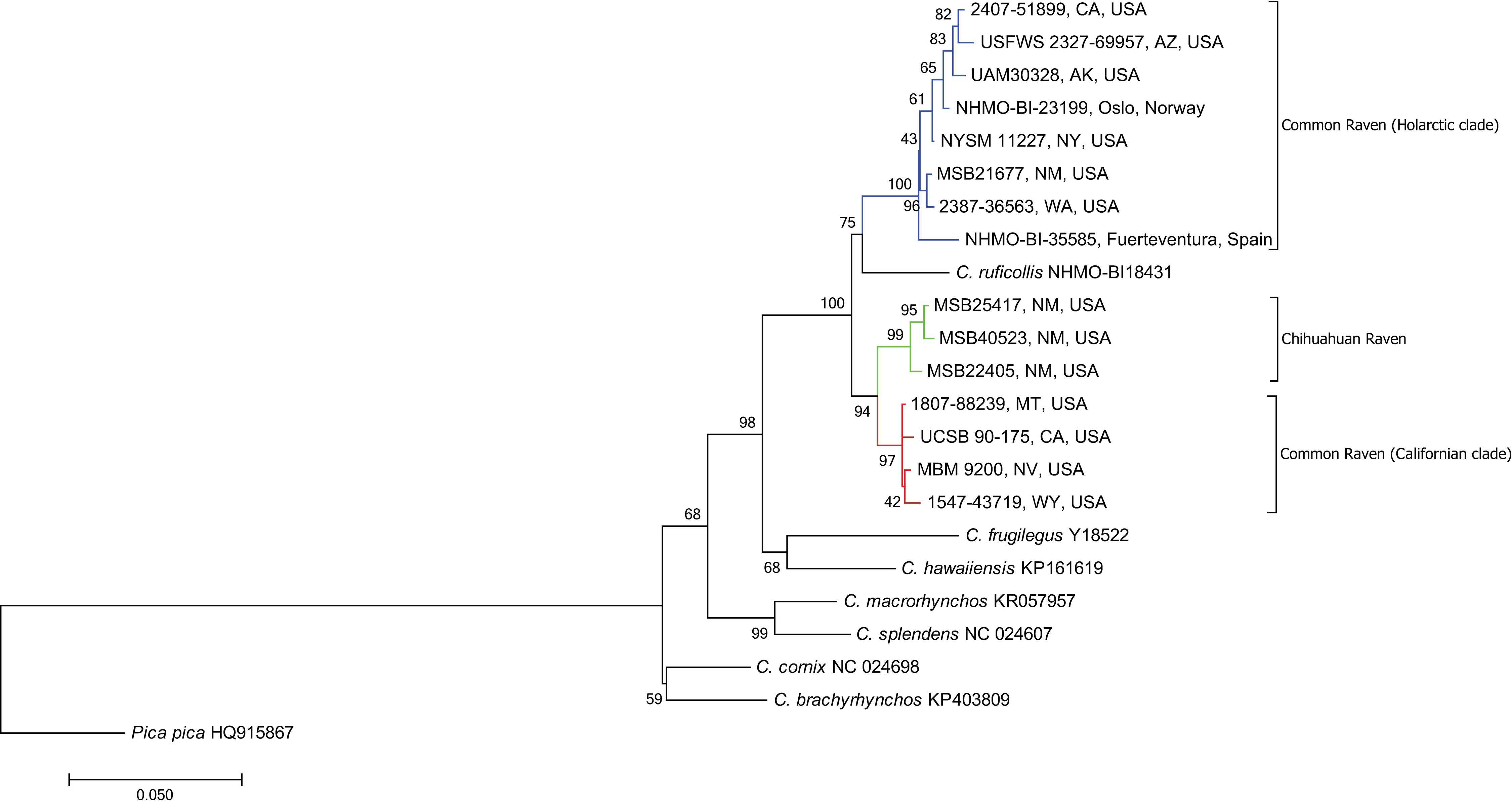

Supplement: S4 Fig — C moriorum was excluded from the tree due to missing data. The tree was rooted with Pica pica. USA = United States of America, CA = California, AZ = Arizona, AK = Alaska, NY = New York, NM = New Mexico, WA = Washington, WY = Wyoming, MT = Montana, NV = Nevada. (TIF) [file pone.0187316.s004.tif]
