# Supplementary material for: Sequencing of the complete mitochondrial genome of the common raven Corvus corax (Aves: Corvidae) confirms mitogenome-wide deep lineages and a paraphyletic relationship with the Chihuahuan raven C. cryptoleucus
Source: PLoS One. 2017 Oct 30;12(10):e0187316. doi: 10.1371/journal.pone.0187316 (PMC5662180; doi:10.1371/journal.pone.0187316)
Supplement: S1 Table — The table provide index of substitution saturation (ISS) and critical values of ISS for symmetric and asymmetric tree topologies. The ISS values were significantly lower (p <0.0001) than the critical ISS in all data sets. (DOCX) [file pone.0187316.s005.docx]

S1 Table. Test of mutational saturation in the five data sets with the 13 mitochondrial protein coding genes. The table provide index of substitution saturation (I_SS_) and critical values of I_SS_ for symmetric and asymmetric tree topologies. The I_SS_ values were significantly lower (p <0.0001) than the critical I_SS_ in all data sets.

|  |  | Critical I_SS_^a^ | |
| --- | --- | --- | --- |
| Data set | I_SS_ | Symmetric | Asymmetric |
| codon position 1,2,3 | 0.027 | 0.849 | 0.677 |
| codon position 1,2 | 0.018 | 0.840 | 0.681 |
| codon position 1 | 0.006 | 0.825 | 0.664 |
| codon position 2 | 0.031 | 0.825 | 0.664 |
| codon position 3 | 0.049 | 0.825 | 0.664 |
| ^a^Critical value at which the sequences will begin to fail to recover the true tree . | | | |
