# Supplementary material for: Sequencing of the complete mitochondrial genome of the common raven Corvus corax (Aves: Corvidae) confirms mitogenome-wide deep lineages and a paraphyletic relationship with the Chihuahuan raven C. cryptoleucus
Source: PLoS One. 2017 Oct 30;12(10):e0187316. doi: 10.1371/journal.pone.0187316 (PMC5662180; doi:10.1371/journal.pone.0187316)
Supplement: S2 Table — (DOCX) [file pone.0187316.s006.docx]

S2 Table. Accession number, total number of reads, number of reads after trimming, reads assembled in the iterative mapping, average coverage in MITObim assembly, GenBank accession numbers and GenBank BioSample ID for each sample.

| Acc. No. | Total reads | Reads surviving trimming (%) | Reads mapped to mitogenome (% of survived reads) | Avg. coverage (X) in MITObim assembly | GenBank acc.no. | GenBank BioSample ID |
| --- | --- | --- | --- | --- | --- | --- |
| 2407-51899 | 26,363 | 15,125 (57.6) | 13,925 (92.1) | 225.08 | KX245135 | SAMN04978507 |
| USFWS 2327-69957 | 23,745 | 13,328 (56.1) | 12,155 (91.2) | 180.91 | KX245136 | SAMN04995030 |
| UAM30328 | 10,568 | 6,006 (57.1) | 5,728 (95.4) | 115.7 | KX245146 | SAMN04995031 |
| NYSM 11227 | 25,146 | 13,871 (54.9) | 12,913 (93.1) | 223.53 | KX245148 | SAMN04995032 |
| MSB21677 | 28,717 | 14,880 (51.9) | 13,469 (90.5) | 236.91 | KX245133 | SAMN04995029 |
| 2387-36563 | 37,700 | 20,831 (55.4) | 18,298 (87.8) | 284.34 | KX245134 | SAMN04995033 |
| NHMO-BI-23199 | 38,609 | 22,241 (57.4) | 19,491 (87.6) | 282.36 | KX245145 | SAMN04995034 |
| NHMO-BI-35585 | 31,093 | 16,162 (52.3) | 15,316 (94.8) | 264.77 | KX245141 | SAMN04995035 |
| 1547-43719 | 33,406 | 19,116 (57.3) | 16,672 (87.2) | 257.77 | KX245140 | SAMN04995036 |
| UCSB 90-175 | 24,235 | 13,359 (55.2) | 11,874 (88.9) | 206.42 | KX245142 | SAMN04995037 |
| 1807-88239 | 29,443 | 16,904 (57.5) | 15,154 (89.6) | 226.43 | KX245137 | SAMN04995038 |
| MBM 9200 | 30,641 | 17,175 (56.3) | 15,479 (90.1) | 244.76 | KX245138 | SAMN04995039 |
| MSB25417 | 31,212 | 17,545 (56.2) | 13,607 (77.6) | 220.03 | KX245139 | SAMN04995040 |
| MSB40523 | 23,379 | 13,269 (56.4) | 12,334 (93) | 185.62 | KX245147 | SAMN04995041 |
| MSB22405 | 32,608 | 17,372 (53.4) | 15,599 (89.8) | 252.86 | KX245143 | SAMN04995042 |
| NHMO-BI-18431 | 24,002 | 13,931 (58.23) | 12,500 (89.7) | 185.52 | KX245144 | SAMN04995043 |
